# Supplementary material for: Di-(2-ethylhexyl) Phthalate Triggers Proliferation, Migration, Stemness, and Epithelial–Mesenchymal Transition in Human Endometrial and Endometriotic Epithelial Cells via the Transforming Growth Factor-β/Smad Signaling Pathway
Source: Int J Mol Sci. 2022 Apr 1;23(7):3938. doi: 10.3390/ijms23073938 (PMC8999884; doi:10.3390/ijms23073938)
Supplement: Supplementary file 1 [file ijms-23-03938-s001.zip › ijms-1642139-supplementary.pdf]

International Journal of
Molecular Sciences

Supplementary Materials

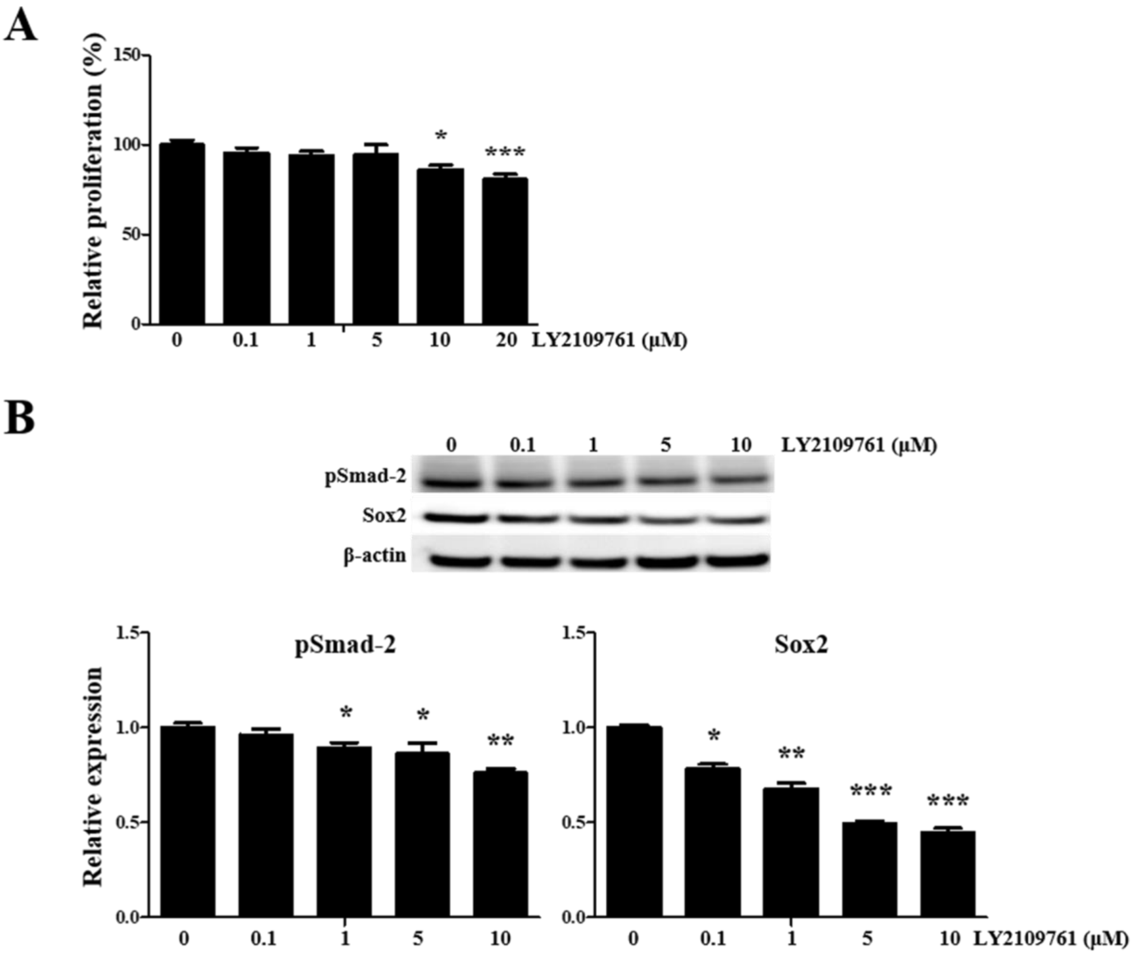

Figure S1. The selective TGF-βR1/2 inhibitor LY2109761 inhibits cell proliferation by pSmad-2 suppression in human endometrial epithelial cells. (A) LY2109761 (0.1 to 20 μM) displays cellular toxicity dose-dependently. (B) LY2109761 (0.1 to 10 μM) represses pSmad-2 and Sox2 protein levels, as detected using western blot analysis. β-actin was used as the internal loading control for data normalization. All data are expressed as relative values against their respective control group. Data represent the mean ± standard deviation of three independent experiments. * $p < 0.05$, ** $p < 0.01$, and *** $p < 0.001$.
